# Clinicopathological Association of Autophagy Related 5 Protein with Prognosis of Colorectal Cancer

**DOI:** 10.3390/diagnostics11050782

**Published:** 2021-04-26

**Authors:** Wan-Hsiang Hu, Wen-Chi Yang, Pei-Feng Liu, Ting-Ting Liu, Paul Morgan, Wei-Lun Tsai, Hung-Wei Pan, Cheng-Hsin Lee, Chih-Wen Shu

**Affiliations:** 1 Department of Colorectal Surgery, Kaohsiung Chang Gung Memorial Hospital and Chang Gung University College of Medicine, Kaohsiung 83341, Taiwan; gary.hu0805@gmail.com; 2 Graduate Institute of Clinical Medical Science, College of Medicine, Chang Gung University, Kaohsiung 83341, Taiwan; 3 Division of Hematology and Medical Oncology, Department of Internal Medicine, E-DA Hospital, Kaohsiung 82445, Taiwan; wenchi890079@gmail.com; 4 School of Medicine for International Students, I-Shou University, Kaohsiung 82445, Taiwan; buddymorgan@gmail.com (P.M.); d89444001@gmail.com (H.-W.P.); 5 Department of Biomedical Science and Environmental Biology, Kaohsiung Medical University, Kaohsiung 80708, Taiwan; pfliu908203@gmail.com (P.-F.L.); angioadsc@gmail.com (C.-H.L.); 6 Department of Medical Research, Kaohsiung Medical University Hospital, Kaohsiung 80708, Taiwan; 7 Department of Medical Laboratory Science, I-Shou University, Kaohsiung 82445, Taiwan; liutt107@cgmh.org.tw; 8 Department of Pathology, Kaohsiung Chang Gung Memorial Hospital and Chang Gung University College of Medicine, Kaohsiung 83341, Taiwan; 9 Department of Internal Medicine, Kaohsiung Veterans General Hospital, Kaohsiung 81362, Taiwan; wltsai@vghks.gov.tw; 10 School of Medicine, National Yang-Ming University, Taipei 11221, Taiwan; 11 Institute of Biopharmaceutical Sciences, National Sun Yat-sen University, Kaohsiung 80424, Taiwan

**Keywords:** autophagy, ATG5, prognosis, colorectal cancer

## Abstract

Gene mutation and pathogenesis bacteria are highly associated with colorectal cancer (CRC) development and progression. Autophagy is a self-clearance pathway to degrade abnormal proteins and infected bacteria in cells. Autophagy plays a dual role in cancer development. Among the autophagy-related (ATG) proteins, ATG5 is the key component required for the core machinery of autophagy. However, the role of ATG5 in CRC malignancy remains unclear. Herein, we found that a high ATG5 protein level was correlated with poor overall survival (OS) and disease-free survival (DFS) of 118 patients with CRC. After stratification with demographic and clinicopathologic factors, a high ATG5 protein level was significantly correlated with unfavorable overall survival in female and elder (>60 year) CRC patients and tumor tissues with poor differentiation, late T stages (III + IV), whereas the ATG5 protein level was positively associated with the overall survival in CRC patients without lymph node invasion and radiation therapy. In contrast, a high ATG5 protein level was significantly associated with worse DFS in CRC patients with early stage of AJCC and no radiation therapy. In addition, colorectal cancer cells stably harboring small interfering RNA (siRNA) against ATG5 diminished the tumorsphere formation and sensitized cancer cells to chemotherapeutic agents. Taken together, our results suggest that ATG5 might be a prognostic biomarker for CRC and a potential therapeutic target for CRC patients.

## 1. Introduction 

Colorectal cancer (CRC) is the top three leading cause of cancer death in both men and women worldwide, particularly in developed countries [1]. CRC cancer related mortality has been increased by almost 50% in over the past 50 years and an over 10% mortality increase is expected by 2030, resulting in more than 13 million deaths worldwide [2]. The CRC incidence is largely increased, which is largely attributed to the increase in population ageing, poor dietary habits, smoking, insufficient physical activity, and obesity [2]. Regarding the potential mechanisms of tumorigenesis and malignancy of CRC, gene mutation, epigenetic change and pathogenesis bacteria are involved in the progression of CRC [3,4]. 

Autophagy is a cellular pathway that reacts under environmental stimulus to maintain homeostasis by degrading abnormal cytoplasmic components including pathogenesis bacteria, organelles, lipids, and proteins [5,6]. These cellular components are digested by lysosomal lytic enzymes for recycling these abnormal components during autophagy [5]. Macroautophagy, microautophagy, and chaperone-mediated autophagy are the three most common types of mammalian autophagy [5]. However, the major pathway by which cytoplasmic proteins and organelles are degraded is believed to occur by means of macroautophagy [5]. Autophagy has been implicated in many cancers for its duality in tumor progression [5,6]. On one end, autophagy plays a critical role in the suppression of tumor progression [5,6]. Alternatively, autophagy has also been shown to promote the survivability and progression of tumors under unfavorable conditions. 

To date, more than 41 mammalian autophagy-specific regulatory genes have been discovered [7,8,9]. Of them all, ATG5 is a key regulator of autophagosome formation and is considered an essential protein for the induction of autophagy [5,10]. ATG5 is a key regulator of the switch between autophagy and apoptosis [10]. However, the role of ATG5 in cancer has been inadequately investigated [10]. Several studies have shown that the suppression of ATG5 is connected to benign adenomas and the progression of benign tumors to cancer is ultimately a consequence of a defect in autophagy [11]. In CRC, ATG5 depletion can either inhibit or promote tumor growth [2]. 

Herein, we investigated the association of the ATG5 protein level with the overall survival and disease-free survival in patients with colorectal cancer. We also elucidated the clinical relevance of ATG5 protein levels on sex, age and differentiation. Finally, we assessed the clinical relevance of ATG5 protein levels on the pathological stages and therapy with overall survival in colorectal cancer patients. Our results support the oncogenic role of ATG5 in CRC and suggest that ATG5 could serve as biomarkers or even a therapeutic target for the treatment of CRC.

## 2. Experimental Procedure

### 2.1. Immunohistochemistry (IHC)

In total, 118 tissues were obtained from the Kaohsiung Chang Gung Memorial Hospital as described previously [12]. The Institutional Review Board approved this study, which complies with the Declaration of Helsinki (institutional review board (IRB) number: 201600132B0). Surgical specimens were fixed in a 10% formalin solution and embedded in paraffin. Immunohistochemical stains were performed by using standard reagents and techniques on an i6000 Automated Staining System (BioGenex, San Ramon, CA, USA). The sections were incubated with primary antibodies followed by Ultra Vision Quanto Detection System kits (Thermo Fisher Scientific, Fremont, CA, USA). The primary antibodies used were anti-ATG5 (Clone EPR1755(2), 1:200; Abcam, Bristol, UK). Positive and negative controls were done according to manufacturer’s instruction. The colored immunohistochemistry (IHC) stains for each protein were developed at room temperature and counterstained with hematoxylin.

### 2.2. Evaluation of IHC

The slides were evaluated by one pathologist blind to clinicopathologic data. The H-score method was adopted to assign a continuous score to each patient based on the percentage of cells at different staining intensities. The percentages of tumor cells with detectable cytoplasmic immunoreactivity for ATG5 were recorded using a 5% increment. The labeling intensity was given a score from 0 to 3, corresponding to non-detectable, weak, moderate and strong staining, respectively. The H-score was defined as the product of the percentage of immunoreactive positive tumor cells and the labeling intensity. Obviously, the index could range from 0 to 300, with 300 corresponding to all (100%) tumor cells displaying strong (3) staining. The final score of each tissue was calculated as intensity multiplied by (percentage × 100), ranging from 0 to 300. For survival analysis, the ATG5 protein levels were categorized into low and high, using the cutoff based on the receiver operating characteristic (ROC) curve. 

### 2.3. Cell Culture and Stable Transfection

The human colorectal cancer HCT116 cells or human embryonic kidney cells HEK293T were cultured with DMEM (Gibco, Life Technologies, CA, USA) and 10% fetal bovine serum (FBS) in a CO2 incubator at 37 °C. The HEK293T cells were reversely transfected with nontargeting shRNA or shRNA against ATG5 (TRCN0000151963, obtained from the RNAi Consortium in Taiwan as previously described) [13,14]. The viral supernatant containing shRNA was harvested, HEK293T cell debris was removed for further infection into HCT116 cells, and stable clones were selected using puromycin (2 μg/mL). The knockdown efficiency was determined with immunoblotting using primary antibody against ATG5 (Clone EPR1755(2), Abcam, Bristol, UK) and ACTB (β-actin, A5441) (Sigma-Aldrich, St. Louis, MI, USA) and HRP-labeled secondary antibodies (Santa Cruz Biotechnology, Dallas, TX, USA, sc-2004 or sc-2005). The protein levels were detected by ChemiDoc XRS Imaging System (Bio-Rad, Hercules, CA, USA).

### 2.4. Sphere Culture and Live/Dead Assay

HCT116 is a colorectal cancer cell line with Kras and β-catenin mutations [15,16], which induces cancer stem cells-like markers, such as CD133, CD44, and CD166 [17]. In addition, according to our previous reports, HCT116 is easy to grow in a spheroid cell culture model [13,14,18]. Thus, HCT116 cells were used for sphere culture model in this study. The HCT116 cells harboring shRNA (4000 cells/well) were cultured in a 96-well plate (ultra-low attachment, Costar^®^, Sigma-Aldrich Corp. St. Louis, MO, USA) for at least overnight until tumorsphere formation. The spheroid cells were treated with doxorubicin (Dox, Selleckchem, Houston, TX, USA) for 48 h. The living cells were stained with Calcein AM (1 μM) and the dead cells were stained by Ethidium homodimer-1 (EthD-1, 2 μM) (LIVE/DEAD^®^ Viability/Cytotoxicity Kit, ThermoFisher Scientific) for 30 min. The cells were observed by fluorescence microscopy and the live/dead cell population was quantitated with a Fluoroskan Ascent FL reader (Thermo Fisher Scientific) using an excitation at 485 nm and emissions at 530 nm and 645 nm.

### 2.5. Statistical Analysis

The protein level of ATG5 in tumor tissues was analyzed for its association with cumulative survival curves using Kaplan–Meier method and the significance was accessed by the log-rank test. The association of the ATG5 protein level with the overall survival or disease-free survival (DFS) of colorectal cancer patients was analyzed by a multivariate Cox regression model with adjustment for cell differentiation (moderate + poor vs. well) and AJCC pathological stage (stage III + IV vs. stage I + II). A two-sided value of *p* < 0.05 was considered as statistically significant. For cell culture experiments, since our results are from at least three independent experiments and a normal distribution is not expected, the significant results were calculated by a non-parametric 2-tailed Student’s *t*-test.

## 3. Results 

**Figure 1:**
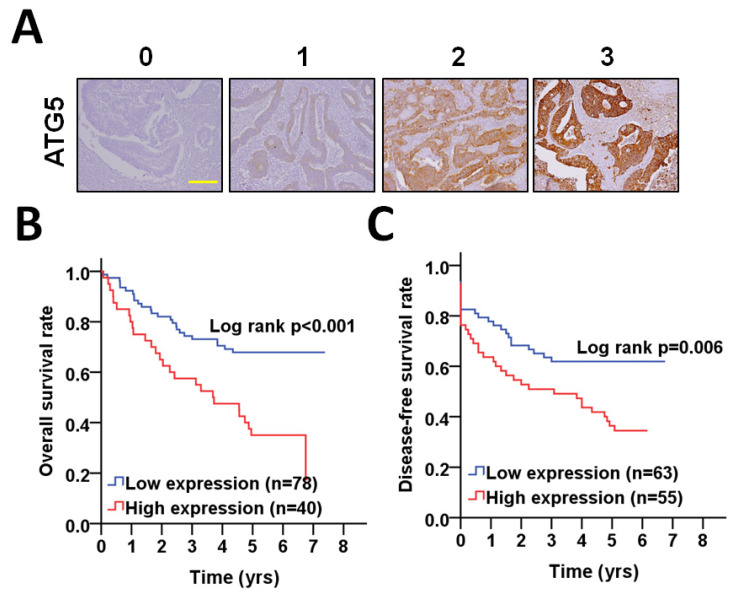
The correlation of the ATG5 protein level on tumor tissues with the overall survival and DFS in patients with CRC. (**A**) The protein level of ATG5 in tumor tissues were determined by immunohistochemistry and the staining intensity was categorized into four degrees as representative images. Scale bar: 200 μm. (**B**) The association of higher (red) or lower (blue) ATG5 protein level on tumor tissues with the overall survival of CRC patients was analyzed by Kaplan–Meier plots. (**C**) The association of higher (red) or lower (blue) ATG5 protein level on tumor tissues with DFS of CRC patients was examined by Kaplan–Meier analysis. The cutoff values to differentiate the high or low ATG5 protein levels in tumor tissues were based on the receiver operating characteristic (ROC) curve.

The expression of ATG5 was initially verified with IHC staining in tumor tissues of CRC patients. Representative staining slides for ATG5 are shown in Figure 1A. The scores for protein levels were categorized into four groups according to the staining intensity (0, no signal; 1, mild; 2, moderate; and 3, strong) and percentage of positive staining (0–100%). The final score of each tissue was calculated as intensity multiplied by (percentage × 100), ranging from 0 to 300. To determine whether ATG5 could be used as biomarkers for prognosis of CRC, the relationship of ATG5 with the overall survival and disease-free survival (DFS) was initially examined by a Kaplan–Meier curve analysis (Figure 1B,C). The results showed that a higher ATG5 expression was associated with a worse overall survival (*p* < 0.001) and DFS (*p* = 0.006) in CRC (Figure 1). Through multiple Cox regression analyses with adjustments for cell differentiation (moderate + poor vs. well) and AJCC pathological stage (stage III + IV vs. stage I + II), patients with higher ATG5 expression had shorter DFS in both CRC (AHR: 2.76, 95% CI: 1.49–4.82, *p* < 0.001) and DFS (AHR: 2.11, 95% CI: 1.25–3.54, *p* = 0.005, Table 1). These results imply that the ATG5 protein plays a role in the overall survival and DFS of CRC patients. 

**Figure 2:**
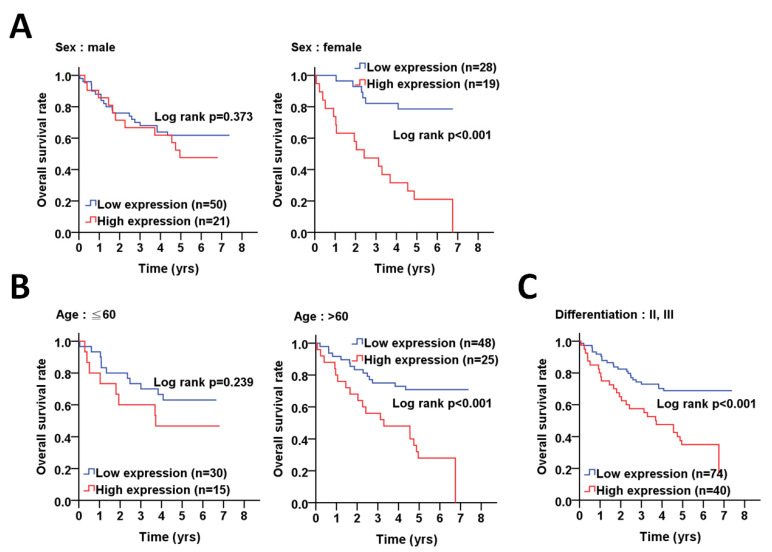
The clinical relevance of ATG5 protein levels on sex, age and differentiation with overall survival in colorectal cancer patients. (**A**) Kaplan–Meier plots were used for the stratified analysis to determine the association of ATG5 protein level with the overall survival based on sex, (**B**) age (60 years old) and (**C**) differentiation. The cutoff values for the high or low ATG5 protein levels in tumor tissues were selected according to the ROC curve. The significance of ATG5 protein levels on survival is shown with log rank.

**Figure 3:**
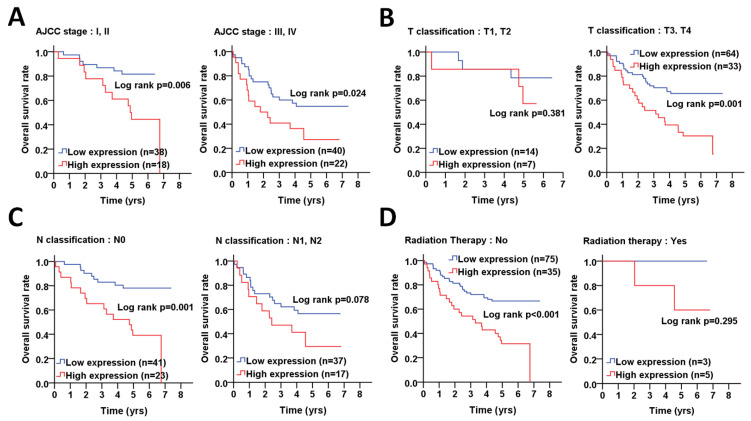
The correlation of ATG5 protein levels with the overall survival in CRC patients with certain pathological stages and radiation therapy. (**A**) The CRC patients were stratified into early and advanced pathological stages, including AJCC, (**B**) tumor size (T stages), (**C**) lymph nodes invasion (N stages), and (**D**) radiation therapy. The association of the ATG5 protein level in tumor tissues with the overall survival was analyzed by Kaplan–Meier plots. The ROC curve was used to determine the cutoff values to differentiate the high (red) or low (low) ATG5 protein levels in tumor tissues. The log rank was used to determine the significance of ATG5 protein levels on the overall survival of CRC patients.

We further stratified the ATG5 expression with the overall survival according to different clinicopathological features and found that a high expression of ATG5 was associated with a poor overall survival in females (*p* < 0.001, Figure 2A), elderly patients (>60) (*p* < 0.001, Figure 2B), poorly differentiated patients (*p* < 0.001, Figure 2C). Moreover, a high ATG5 protein level was significantly correlated with the overall survival in CRC patients with AJCC pathological stages I + II (*p* = 0.006, Figure 3A) and III + IV (*p* = 0.024), particularly in CRC patients with advanced T stage T3 + T4 (*p* = 0.001, Figure 3B) and without lymph node invasion N0 (*p* = 0.001, Figure 3C). Interestingly, a higher ATG5 expression had a worse overall survival rate in CRC patients without radiation therapy (*p* < 0.001, Figure 3D). 

In addition, multiple Cox regression analyses showed that ATG5 was associated with poor overall survival in females (AHR: 6.61, *p* < 0.001, Table 2), elderly patients (>60) (AHR: 4.13, *p* < 0.001), poorly differentiated patients (AHR: 2.75, *p* < 0.001), patients with AJCC pathological stages I + II (AHR: 3.79, *p* = 0.010), III + IV (AHR: 2.25, *p* = 0.021), and T3 + T4 (AHR: 2.77, *p* = 0.001) and CRC patients without radiation therapy (AHR: 3.29, *p* < 0.001). 

**Figure 4:**
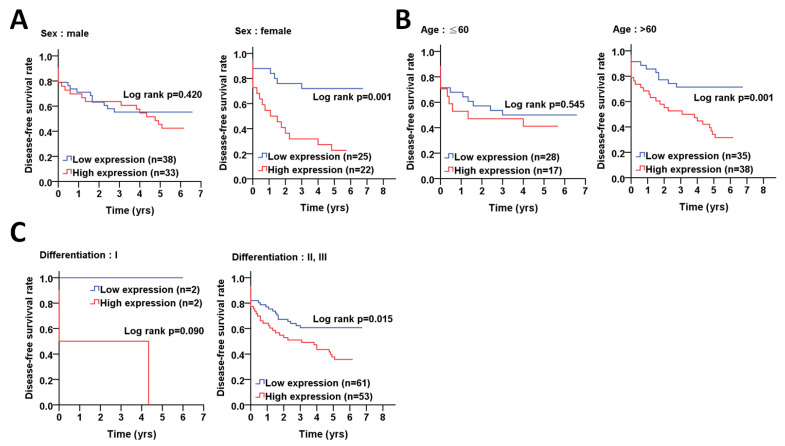
The clinical relevance of ATG5 protein levels on sex, age and differentiation with disease-free survival in colorectal cancer patients. (**A**) Kaplan–Meier plots were used for the stratified analysis to determine the association of ATG5 protein level with disease-free survival based on sex, (**B**) age (60 years old), and (**C**) differentiation. The cutoff values for the high or low ATG5 protein levels in tumor tissues were determined according to the ROC curve. The significance of ATG5 protein levels on survival is shown with log rank.

**Figure 5:**
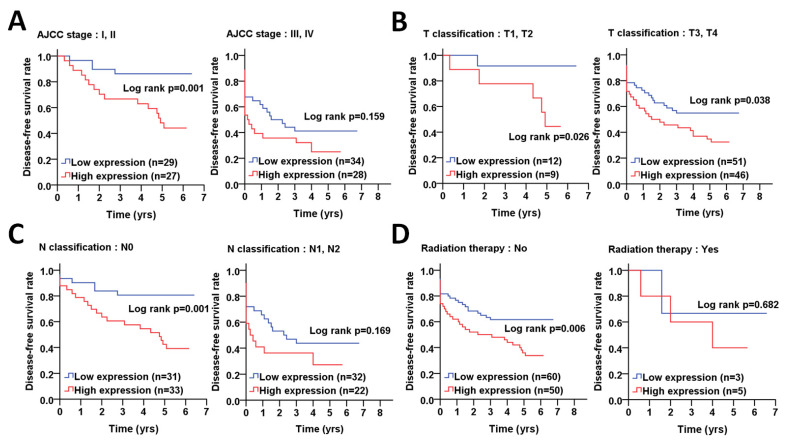
The correlation of ATG5 protein levels with DFS in CRC patients with certain pathological stages and radiation therapy. (**A**) The CRC patients were stratified into early and advanced pathological stages, including AJCC, (**B**) tumor size (T stages), (**C**) lymph nodes invasion (N stages), and (**D**) radiation therapy. The association of ATG5 protein level in tumor tissues with DFS was analyzed by Kaplan–Meier plots. The ROC curve was used to determine the cutoff values to differentiate the high (red) or low (low) ATG5 protein levels in tumor tissues. The log rank was used to determine the significance of ATG5 protein levels on DFS of CRC patients.

To determine whether ATG5 is correlated with relapse in certain groups of CRC, we further stratified the CRC patients according to clinicopathological factors to analyze the association of ATG5 protein levels with DFS using the Kaplan–Meier curve (Figure 4). The results showed that high protein levels of ATG5 were notably associated with a shorter DFS in female (*p* = 0.001) and elderly CRC (*p* = 0.001), poorly differentiated patients (*p* = 0.015, Figure 4A–C). Moreover, a high ATG5 protein level was significantly correlated with DFS in CRC patients with early AJCC pathological stages I + II (*p* < 0.001, Figure 5A), T stage T1 + T2 (*p* = 0.025) and T3 + T4 (*p* = 0.038, Figure 5B) and without lymph node invasion N0 (*p* = 0.001, Figure 3C). Similarly, a higher ATG5 expression had an unfavorable DFS in CRC patients without radiation therapy (*p* = 0.006, Figure 5D). 

After adjustment with cell differentiation (moderate + poor vs. well) and/or pathological stage (advanced stage vs. early stage), multiple Cox regression analyses indicated that ATG5 was associated with poor DFS in females (AHR: 3.75, *p* = 0.004, Table 3), elderly patients (>60) (AHR: 3.18, *p* = 0.002), poorly differentiated patients (AHR: 1.99, *p* = 0.010), patients with AJCC pathological stages I + II (AHR: 5.09, *p* = 0.004), T3 + T4 (AHR: 1.89, *p* = 0.023) and CRC patients without lymph node invasion (AHR:3.74, *p* = 0.005) and radiation therapy (AHR: 3.29, *p* < 0.001). These results suggested that the ATG5 might be crucial in tumor relapse in patients with CRC. Similarly, a Cox regression analysis with adjustments for cell differentiation and AJCC pathological stage showed that an increased protein level of ATG5 had a higher hazard ratio in patients with CRC (AHR: 1.95, *p* = 0.045, Table 3).

**Figure 6:**
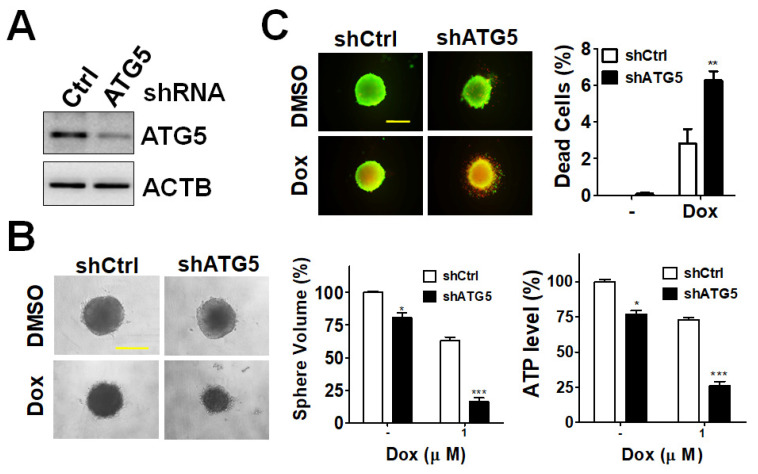
Effects of silencing ATG5 on chemosensitivity in tumorspheres. (**A**) HCT116 cells were transfected with scrambled shRNA or shRNA against ATG5 and selected for stable clones. The knockdown efficiency of ATG5 in HCT116 were confirmed with immunoblotting. (**B**) HCT116 cells carrying scrambled shRNA or shRNA against ATG5 were cultured to form spheres. The tumorspheres were treated with DMSO or Dox (1 μM) for 48 h to image the spheres. Scale bar: 400 μm (left panel). HCT116 spheroid cells were lysed to assess cell viability (right panel). (**C**) The tumorspheres were treated with Dox (1 μM) for 48 h. The viable (green) and dead (red) spheres were observed under fluorescent microscope. The quantified results are expressed as the mean ± SEM from three independent experiments (n = 6). * *p* < 0.05, ** *p* < 0.01, *** *p* < 0.001.

Given the clinical results mentioned above, the ATG5 protein level was positively correlated with the relapse of CRC as mentioned above, implying that the ATG5 may be involved in the cancer stemness and drug resistance characteristics of cancer cells. The tumorsphere culture can mimic the oxygen and nutrients deprivation situation in vivo [19]. The colorectal cancer cell line HCT116 expressed more cancer stem cells-like marker CD133, which is easier to grow as a tumorsphere, as reported previously [20]. Thus, the tumorsphere formation was used to determine the potential function of ATG5 in CRC. The CRC HCT116 cells were silenced with scrambled shRNA or shRNA against ATG5 (Figure 6). The knockdown efficiency in colorectal cancer HCT116 cells was confirmed by immunoblotting (Figure 6A). Silencing the ATG5 significantly inhibited size and viability of the tumorsphere in HCT116 cells (Figure 6B). Moreover, the dead cell population was increased in HCT116 silenced with shRNA against ATG5 when exposed to doxorubicin compared to that with scrambled shRNA, indicating that ATG5 silenced cells were sensitive to the chemotherapeutic agent (Figure 6C). These results imply that the ATG5 might facilitate the cell proliferation and drug resistance of CRC cells.

## 4. Discussion 

Among the mammal ATG proteins, ATG5 is one of the most studied for the role of autophagy in various diseases. Defects in autophagy are linked to many inflammatory diseases [21], including cancer [22]. Full-length *ATG5* mRNA expression is lower in tumor tissues in several cancer types compared to normal tissues [23]. Somatic ATG5 splice mutations lose the full-length expression and impair the LC3-II formation. However, the liver-specific knockout of ATG5 develops to benign liver tumors in a high penetrance mouse model [24]. Thus, the association of ATG5 protein in prognosis and the function in colorectal cancer remains unclear. Herein, we report the following findings: First, a high ATG5 protein level was significantly associated with a worse overall survival and DFS. Second, a stratification analysis indicated that high ATG5 protein levels were correlated with poor overall survival and DFS in female and elderly (>60 yr) patients. Third, a high ATG5 protein level was positively correlated with the overall survival and DFS in patients with large tumor size (T3 and T4) or patients without lymph node invasion and radiotherapy. Fourth, silencing the ATG5 diminished the tumorsphere formation and sensitized colorectal cancer cells to chemotherapeutic agents. Our study suggested that the ATG5 protein might promote tumor progression and malignancy, which provide the ATG5 as a prognostic marker for patients with colorectal cancer. 

The role of autophagy in cancer progression and drug resistance has been studied previously [25]. Autophagy promotes cell proliferation and survival in the central part of a tumor with limited nutrients and oxygen or in metastatic cancer cells [26]. Autophagy is induced to protect cancer cells from damages of radiation [22] or chemotherapeutic agents [14]. Moreover, cancer patients with early AJCC stage or without lymph node invasion and radiotherapy usually have better overall survival and disease-free survival. Our present results suggest that a high ATG5 was associated with poor survival in colorectal cancer patients with early stages. In line with our study, the ATG5 level is crucial for tumorigenesis of pancreatic cancer cells with oncogenic Kras [27], implying ATG5 may facilitate early tumor development in colorectal cancer. Besides, only patients with rectal cancer were treated with radiotherapy and the sample size (n = 8) was not enough to obtain statistical significance. Thus, the association of the ATG5 protein in colorectal patients treated with radiotherapy require a bigger cohort to evaluate. 

Clinical association of ATG genes with cancer patients is also reported in various cancer types. The Cancer Genome Atlas (TCGA) data analysis for 63 ATG genes indicates that 7 ATG genes are significantly associated with PD-L1 expression and poor survival in patients with hepatocellular carcinoma [28]. Similar, a TCGA data analysis for 234 ATG genes in patients with prostate cancer reveals that 5 ATGs (FAM215A, FDD, MYC, RHEB, and ATG16L1) can be used as a poor prognostic autophagy signature for overall survival, whereas 22 ATG genes (ULK2, NLRC4, MAPK1, ATG4D, MAPK3, ATG2A, ATG9B, FOXO1, PTEN, HDAC6, PRKN, HSPB8, P4HB, MAP2K7, MTOR, RHEB, TSC1, BIRC5, RGS19, RAB24, PTK6, and NRG2) can be used as a signature model for unfavorable DFS [29]. Moreover, among 149 ATG genes, 22 ATG gene signature is significantly associated with shorter overall survival in patients with non-small cell lung cancer (NSCLC) [30]. These results suggest that autophagy is highly associated with cancer progression and malignancy. However, these finding are based on an mRNA level for their clinical correlation. Herein, our results showed that high ATG5 protein levels might facilitate tumor progression and survival, which, in turn, might result in worse overall survival and recurrence. 

In terms of ATG gene mutation on its expression, large-scale screening results of gene mutation for numerous cancer types indicate that ATG genes expression are unchanged in most of cancer types [31]. Although the somatic point mutations of the ATG5 are identified in multiple cancer types, including patients of cervical cancer, prostate cancer, colorectal cancer, and hepatocellular carcinoma [23], an ATG5 mutation has no effects on the full length mRNA expression in colorectal cancer. Nevertheless, the full length ATG5 mRNA and protein levels are decreased in tumor tissues compared to normal tissues in patients with colorectal cancer [10,23]. In contrast, our results showed that high ATG5 protein levels had unfavorable consequences for the overall survival and DFS. Taken together, these results suggest that the ATG5 may serve as a tumor suppressor in tumor imitation, whereas it serves as a tumor promoter in cancer metastasis and drug resistance of colorectal cancer. Besides, some of the mutations in ATG5 genes found in the DU145 prostate cancer cell line may interfere its functions, such as binding with ATG16L1 and ATG5-ATG12 conjugation [23]. Thus, further study may be required for the mutation and function of ATG5 in tumor tissues of colorectal cancer. 

Though the role of ATG5 is like a double-edged sword in cancer development and progression, the ATG5 knockout causes benign tumors in aged mice (19 month) [24]. Our results also indicated that a high level of ATG5 was associated with poor prognosis. In addition, silencing the ATG5 diminished tumor sphere formation and sensitized colorectal cancer cells to chemotherapeutic agents, suggesting that ATG5 could be a therapeutic target for colorectal cancer. 

## Figures and Tables

**Table 1:** Impact of ATG5 expression levels on survival of CRC patients.

Variable		No. (%)	CHR (95% CI)	*p* Value *	AHR (95% CI)	*p* Value ^†^
**Overall survival**						
ATG5	Low	78 (66.1)	1.00		1.00	
	High	40 (33.9)	2.58 (1.49–4.45)	0.001	2.76 (1.58–4.82)	<0.001
**Disease-free survival**						
ATG5	Low	63 (53.4)	1.00		1.00	
	High	55 (46.6)	1.98 (1.18–3.32)	0.010	2.11 (1.25–3.54)	0.005

Abbreviations: SCC, squamous cell carcinoma; CHR, crude hazard ratio; CI, confidence interval; AHR, adjusted hazard ratio; AJCC, American Joint Committee on Cancer; RT, radiotherapy. * *p* values were estimated by Cox’s regression. ^†^ *p* values were adjusted for cell differentiation (moderate + poor vs. well) and AJCC pathological stage (stage III + IV vs. stage I+II) by multivariate Cox’s regression.

**Table 2:** Association of the ATG5 protein level on the overall survival according to demographic and clinicopathologic factors in CRC patients.

Variable		No. (%)	CHR (95% CI)	*p* Value *	AHR (95% CI)	*p* Value ^†^
Sex						
Female	Low	28 (59.6)	1.00		1.00	
	High	19 (40.4)	6.25 (2.42–16.13)	<0.001	6.61 (2.54–17.19)	<0.001 ^a^
Male	Low	50 (70.4)	1.00		1.00	
	High	21 (29.6)	1.40 (0.67–2.94)	0.375	1.51 (0.71–3.23)	0.285 ^a^
Age, years						
≦60	Low	30 (66.7)	1.00		1.00	
	High	15 (33.3)	1.72 (0.69–4.28)	0.244	1.64 (0.66–4.08)	0.289 ^a^
>60	Low	48 (65.8)	1.00		1.00	
	High	25 (34.2)	3.36 (1.68–6.73)	0.001	4.13 (1.98–8.58)	<0.001 ^a^
Cell differentiation						
Well	Low	4 (100.0)	1.00		1.00	
	High	0 (0)	Incalculable		Incalculable	
Moderate, poor	Low	74 (64.9)	1.00		1.00	
	High	40 (35.1)	2.66 (1.52–4.65)	0.001	2.75 (1.57–4.80)	<0.001 ^b^
AJCC pathological stage						
I, II	Low	38 (67.9)	1.00		1.00	
	High	18 (32.1)	3.54 (1.35–9.33)	0.010	3.79 (1.37–10.45)	0.010 ^c^
III, IV	Low	40 (64.5)	1.00		1.00	
	High	22 (35.5)	2.14 (1.09–4.21)	0.027	2.25 (1.13–4.45)	0.021 ^c^
T classification						
T1, T2	Low	14 (66.7)	1.00		1.00	
	High	7 (33.3)	2.02 (0.41–10.02)	0.391	2.62 (0.44–15.68)	0.293 ^d^
T3, T4	Low	64 (66.0)	1.00		1.00	
	High	33 (34.0)	2.68 (1.50–4.79)	0.001	2.77 (1.54–5.00)	0.001 ^d^
N classification						
N0	Low	41 (64.1)	1.00		1.00	
	High	23 (35.9)	3.75 (1.64–8.61)	0.002	4.03 (1.70–9.57)	0.002 ^e^
N1, N2	Low	37 (68.5)	1.00		1.00	
	High	17 (31.5)	1.94 (0.92–4.11)	0.084	2.05 (0.96–4.38)	0.065 ^e^
Postoperative RT						
No	Low	75 (68.2)	1.00		1.00	
	High	35 (31.8)	2.76 (1.58–4.81)	<0.001	3.29 (1.85–5.85)	<0.001 ^a^
Yes	Low	3 (37.5)	1.00		1.00	
	High	5 (62.5)	40.74 (0.00–7251250.11)	0.548	21.31 (0.00–10349447.27)	0.647 ^a^

Abbreviations: SCC, squamous cell carcinoma; CHR, crude hazard ratio; CI, confidence interval; AHR, adjusted hazard ratio; AJCC, American Joint Committee on Cancer; RT, radiotherapy. * *p* values were estimated by Cox’s regression. ^†^ *p* values were estimated by multivariate Cox’s regression. ^a^ Adjusted for cell differentiation (moderate + poor vs. well) and AJCC pathological stage (stage III + IV vs. stage I + II). ^b^ Adjusted for AJCC pathological stage (stage III + IV vs. stage I + II). ^c^ Adjusted for cell differentiation (moderate + poor vs. well). ^d^ Adjusted for cell differentiation (moderate + poor vs. well) and N classification (N1, N2 vs. N0). ^e^ Adjusted for cell differentiation (moderate + poor vs. well) and T classification (T3, T4 vs. T1+T2).

**Table 3:** Association of the ATG5 protein level on disease-free survival according to demographic and clinicopathologic factors in CRC patients.

Variable		No. (%)	CHR (95% CI)	*p* Value *	AHR (95% CI)	*p* Value ^†^
Sex						
Female	Low	25 (53.2)	1.00		1.00	
	High	22 (46.8)	3.95 (1.62–9.61)	0.002	3.75 (1.54–9.13)	0.004 ^a^
Male	Low	38 (53.5)	1.00		1.00	
	High	33 (46.5)	1.29 (0.67–2.49)	0.442	1.34 (0.68–2.63)	0.398 ^a^
Age, years						
≦60	Low	28 (62.2)	1.00		1.00	
	High	17 (37.8)	1.26 (0.56–2.84)	0.578	1.24 (0.55–2.80)	0.608 ^a^
>60	Low	35 (47.9)	1.00		1.00	
	High	38 (52.1)	3.05 (1.47–6.34)	0.003	3.18 (1.51–6.70)	0.002 ^a^
Cell differentiation						
Well	Low	2 (50.0)	1.00		1.00	
	High	2 (50.0)	104.94 (0.00–13651960.31)	0.439	57.55 (0.00–2876455.83)	0.463 ^b^
Moderate, poor	Low	61 (53.5)	1.00		1.00	
	High	53(46.5)	1.85 (1.10–3.12)	0.021	1.99 (1.18–3.37)	0.010 ^b^
AJCC pathological stage						
I, II	Low	29 (51.8)	1.00		1.00	
	High	27 (48.2)	5.06 (1.68–15.28)	0.004	5.09 (1.68–15.44)	0.004 ^c^
III, IV	Low	34 (54.8)	1.00		1.00	
	High	28 (45.2)	1.47 (0.80–2.72)	0.219	1.43 (0.77–2.67)	0.256 ^c^
T classification						
T1, T2	Low	12 (57.1)	1.00		1.00	
	High	9 (42.9)	7.85 (0.91–67.35)	0.060	7.98 (0.93–68.86)	0.059 ^d^
T3, T4	Low	51 (52.6)	1.00		1.00	
	High	46 (47.4)	1.70 (0.99–2.92)	0.054	1.89 (1.09–3.27)	0.023 ^d^
N classification						
N0	Low	31 (48.4)	1.00		1.00	
	High	33 (51.6)	3.91 (1.56–9.75)	0.004	3.74 (1.49–9.42)	0.005 ^e^
N1, N2	Low	32 (59.3)	1.00		1.00	
	High	22 (40.7)	1.53 (0.78–3.01)	0.217	1.48 (0.74–2.94)	0.266 ^e^
Postoperative RT						
No	Low	60 (54.5)	1.00		1.00	
	High	50 (45.5)	2.00 (1.17–3.41)	0.011	2.22 (1.30–3.80)	0.004 ^a^
Yes	Low	3 (37.5)	1.00		1.00	
	High	5 (62.5)	1.61 (0.16–15.70)	0.684	1.23(0.11–14.18)	0.868 ^a^

Abbreviations: SCC, squamous cell carcinoma; CHR, crude hazard ratio; CI, confidence interval; AHR, adjusted hazard ratio; AJCC, American Joint Committee on Cancer; RT, radiotherapy. * *p* values were estimated by Cox’s regression. ^†^ *p* values were estimated by multivariate Cox’s regression. ^a^ Adjusted for cell differentiation (moderate + poor vs. well) and AJCC pathological stage (stage III + IV vs stage I + II). ^b^ Adjusted for AJCC pathological stage (stage III + IV vs stage I + II). ^c^ Adjusted for cell differentiation (moderate + poor vs. well). ^d^ Adjusted for cell differentiation (moderate + poor vs. well) and N classification (N1, N2 vs N0). ^e^ Adjusted for cell differentiation (moderate + poor vs. well) and T classification (T3, T4 vs T1+T2).

## Data Availability

Not applicable.
